# Characterization of Lactic Acid Bacteria Isolated from Spontaneously Fermented Sausages: Bioprotective, Technological and Functional Properties

**DOI:** 10.3390/foods12040727

**Published:** 2023-02-07

**Authors:** Ivana Nikodinoska, Giulia Tabanelli, Loredana Baffoni, Fausto Gardini, Francesca Gaggìa, Federica Barbieri, Diana Di Gioia

**Affiliations:** 1 Department of Agricultural and Food Sciences (DISTAL), Alma Mater Studiorum-Università di Bologna, 40127 Bologna, Italy; 2 Interdepartmental Center for Industrial Agri-Food Research, University of Bologna, 47521 Cesena, Italy; 3 Department of Agricultural and Food Sciences (DISTAL), Alma Mater Studiorum-Università di Bologna, 47521 Cesena, Italy

**Keywords:** lactic acid bacteria, starter cultures, fermented sausages, traditional meat products

## Abstract

Fermentation is one of the most ancient strategies to improve safety and extend shelf-life of the products. Starter cultures are mainly represented by lactic acid bacteria (LAB), which may also be bioprotective agents controlling the fermentation process, the native microbiota and pathogen outgrowth. This work aimed to select new LAB strains from spontaneously fermented sausages produced in different areas of Italy, which can be effective as starter cultures and bioprotective agents in fermented salami. The strains, mainly belonging to the *Latilactobacillus sakei* species, were characterized for their ability to inhibit major meat pathogens, the presence of antibiotic resistances and amine production. Moreover, technological performances, such as growth and acidification kinetics at increasing NaCl concentrations, were studied. As a result, new autochthonous *Lat. sakei* strains were obtained, lacking antibiotic resistance, possessing antimicrobial activity against *Clostridium sporogenes*, *Listeria monocytogenes*, *Salmonella* and *Escherichia coli* and with high growth performance under osmotic pressure. These strains have the potential for future application to improve the safety of fermented meats, even under conditions in which chemical preservatives are reduced or eliminated. Moreover, studies on autochthonous cultures are pivotal for guaranteeing specific characteristics of traditional products that represent an important cultural heritage.

## 1. Introduction

Fermented meat products, such as salamis, represent a cultural fingerprint dating back millennia. Traditional salami production in Italy is sometimes carried out on small-scale family-based farms, with in-house production of all raw materials [1]. The artisanal products bring the “taste of tradition” where indigenous microbiota, raw materials and production conditions are the main protagonists [2]. Spontaneous fermentation in artisanal small-scale salami production is led by environmental and meat-indigenous biota, whereas industrial-scale fermentation is standardized with the employment of starter cultures [3]. Fermented salamis are restricted ecological niches, harbouring lactic acid bacteria (LAB), *Staphylococcus, Debaryomyces* and *Penicillium* as the main bacterial and fungi genera. LAB are the most employed starters for several matrices, including meat, due to their technological, functional and safety features [3]. The limited carbon availability and the rich amino acid and lipid content in the meat matrix are the shaping parameters of the LAB metabolic behaviour during the fermentation and late ripening process. The initial sugar fermentation and the successive amino acid catabolism have a significant impact on the hygienic and sensory quality of long-ripened fermented meats [4].

Fast LAB growth and the consequent rapid pH drop are the most desirable technological and safety characteristic of meat starter cultures. The fast acidification in salami manufacturing mainly inhibits spoilage and pathogenic bacteria and contributes to desirable textural properties and sensory profile, due to the coagulation of muscle proteins [5]. Moreover, to ensure the safety of fermented dry-cured salamis, nitrate and nitrite are usually added to the meat before the fermentation process. However, this is raising concern among consumers because of the adverse effects of nitrite on health [6]. 

Fermentation and the consequent biopreservation is one of the most ancient microbial-based strategies to improve product safety and extend shelf-life [7]. Protective cultures used in fermented foods are mainly represented by LAB, which might also be added as starters aimed at controlling both the native microbiota and the pathogen outgrowth [8]. The production of antimicrobial compounds such as organic acids, hydrogen peroxide and lytic agents are some of the antagonistic mechanisms employed by bioprotective bacteria [9], but also the production of bacteriocins, known for their bactericidal effects at a specific concentration, may act against mainly gram-positive bacteria [10]. 

The employment of food grade cultures in Europe is regulated by the General EU Food Law (EU Regulation No. 178/2002). It relies on the obligations of the culture supplier for a careful safety assessment of the newly released products [11]. The safety of new microbial candidate strains should be guaranteed by the study of potentially transmissible antibiotic resistance traits and biogenic amine production [12], although antibiotic resistant strains and biogenic amine producers are often naturally present in foods [13,14]. Therefore, the safety characterization of strains to be employed as starter cultures is a prerequisite for their use. 

In this work, indigenous LAB strains were isolated from spontaneously fermented sausages produced in different areas of Italy and characterized for their bioprotective function, safety assessment and technological performances. The aim was to obtain and characterise LAB strains that could be proposed as starter cultures for fermented sausage production and have, at the same time, antimicrobial activity against potential food pathogens. 

## 2. Materials and Methods

### 2.1. Traditional Spontaneously Fermented Sausages Characterization

Three artisanal, homemade, starter-free salami produced in different Italian regions were considered for isolation of new LAB strains: *Salame Bresciano* (BRE), from the Lombardy region; *Salame Romagnolo* (ROM), from the Emilia Romagna Region; and *Salame Basilicata* (BAS), from the Basilicata Region. The sausages from Basilicata differed from the others in their traditional preservation. In fact, after ripening, they were stored in pots and covered with pork fat. For this reason, they are called “*Salsiccia sotto sugna*” [15]. The samples analysed in this work remained under fat for one month at room temperature before sampling. The main characteristics of these fermented sausages, obtained by the producers, are reported in Table 1. 

All sausages contained pepper and no sugar was added. After aseptically removing the casing, 10 g of fermented sausages were 10-fold diluted with 90 mL of 0.9% (*w*/*v*) NaCl and homogenized in a Lab Blender Stomacher (Seward Medical, London, UK) for 2 min. Decimal dilutions were performed and plated onto selective media. For lactobacilli count, MRS agar (Oxoid, Milan, Italy) plates were used and incubated at 30 °C for 48 h in anaerobic conditions. Staphylococci were counted by surface-plating on Baird-Parker (Oxoid), added with egg yolk tellurite emulsion, with incubation at 30 °C for 48 h. For enterococci counts, Slanetz and Bartley medium (Oxoid) incubated at 42 °C for 48 h was used. Finally, *Enterobacteriaceae* were enumerated by plating in violet red bile glucose agar (Oxoid) at 37 °C for 24 h. The analyses were performed in triplicate.

The determination of water activity (a_w_) and pH was performed in triplicate by using an Aqualab CX3-TE (Labo-Scientifica, Parma, Italy) and a pH meter Basic 20 (Crison Instruments, Barcelona, Spain), respectively.

### 2.2. Isolation of LAB Strains and DNA Extraction

Colonies grown onto MRS plates were randomly picked with sterile loop and single colony lines were streaked onto new MRS agar plates. Each selected colony was then inoculated in 10 mL MRS broth and incubated at 30 °C for 24 h. Next, 1 mL overnight culture was submitted to DNA extraction with the Wizard^®^ Genomic DNA Purification Kit (Promega Italia S.r.l., Milan, Italy). The remaining bacterial culture was centrifuged and from the obtained pellet single strain skim milk stocks were prepared and kept at −80 °C. Manufacturer’s instructions for isolating genomic DNA from gram-positive bacteria were followed, except for an additional lysis step (100 mg/mL of lysozyme, followed by an overnight incubation at 37 °C).

### 2.3. Fingerprinting-Based Clustering and 16S rRNA Identification of Biotypes

Clustering of LAB isolates was performed by Random Amplification of Polymorphic DNA (RAPD)-PCR as described by Di Gioia et al. [16]. Cluster analysis of obtained RAPD profiles was carried out with GelCompar II, 6.6 (Applied Maths, Sint-Martens-Latem, Belgium) using Dice’s coefficient of similarity with the un-weighted pair group method arithmetic averages clustering algorithm (UPGMA). Based on the genotypic clustering, amplification of 16S rRNA gene region of representative isolates was performed, according to Gaggia et al. [17] and then sequenced (MWG, Eurofins genomics). The obtained forward and reverse sequences were edited, and consensus sequences were built using the BioEdit software package. Sequences assignment to species or genera was achieved with the genomic data available on NCBI by BLASTn procedure (https://blast.ncbi.nlm.nih.gov/Blast.cgi?PAGE_TYPE=BlastSearch, accessed on 3 June 2021).

### 2.4. Determination of Antibiotic Susceptibility

Antibiotic susceptibility profiles of LAB were phenotypically determined, using Lact-1 and Lact-2 VetMIC microplates, purchased from the National Veterinary Institute (SVA, Uppsala, Sweden). Briefly, individual colonies were suspended in a sterile glass tube containing 4 mL maximum recovery diluent (Biolife, Milan, Italy) to a turbidity of 1 on the McFarland scale (~1 × 10^8^ CFU/mL). Next, 20 μL from the bacterial suspension was diluted in 10 mL ISO-MRS broth (90% Iso-sensitest IST broth, Oxoid + 10% MRS broth) to obtain a final inoculum of ~5 × 10^5^ CFU/mL. After filling with 100 μL of the final suspension (5 × 10^5^ CFU/mL), VetMIC plates were sealed with provided clear film and incubated at 30 °C for 24–48 h, depending on the growth of the strain in the control wells.

An inverted light microscope was used for results interpretation. MIC was considered as the lowest concentration completely inhibiting visible growth. The resistance of LAB to antibiotics was determined according to the cut-off values reported by the FEEDAP Panel and adopted by EFSA [18] for gentamicin (16 mg/L), streptomycin (64 mg/L), tetracycline (8 mg/L), erythromycin (1 mg/L), clindamycin (1 mg/L), chloramphenicol (4 mg/L), kanamycin (16 mg/L for *Leuconostoc* sp. and 64 mg/L for lactobacilli facultative heterofermentative) and ampicillin (2 mg/L for *Leuconostoc* sp. and 4 mg/L for lactobacilli facultative heterofermentative).

### 2.5. Biogenic Amine Production

The identification of biogenic amines production was performed with the use of the medium proposed by Bover-Cid and Holzapfel [19] and further confirmed by HPLC analysis as described by Tabanelli et al. [20].

### 2.6. Antimicrobial Activity Assay

The potential antagonistic activity of LAB strains was evaluated using different indicator strains (listed in Table 2). The activity of all the cells was tested with a spot-on-the-lawn assay, whereas the potential production of antimicrobial compounds present in the cell-free supernatant was tested with the well-diffusion assay (WDA). For the direct antagonistic activity, 10 µL of fresh cell pellet, from LAB strains previously grown overnight, were spotted onto MRS agar plates and incubated for 24 h at 30 °C. Then, plates were overlaid with 10 mL of 0.8% BHI or RCM soft agar (Oxoid, Milan, Italy) containing 10^5^ CFU/mL *Listeria* spp., *E. coli*, *Salmonella* spp. or *Clostridium* spp., respectively, and incubated as indicated in Table 2. Supernatants from meat-borne LAB strains, grown in MRS broth for 24 h at 30 °C, were obtained by centrifugation at 5000× *g* at 4 °C for 10 min. Unmodified and neutralized supernatants at pH 6.8–7.2 with 0.1–1 M NaOH were used for the WDA. After solidification of MRS, BHA (BHI+1.5% agar) and RCA (RCM+1.5% agar) agar plates inoculated with 10^5^ CFU/mL of the indicator strain, 50 µL of supernatant were inoculated into preformed 6-mm diameter wells. Plates were initially placed for 2 h at 4 °C and further incubated as indicated in Table 2. Both assays were performed in duplicate and the presence of inhibition zones around the spotted cells or around the wells was analysed.

### 2.7. Kinetic Modelling of Microbial Growth and Acidification

Growth and acidification kinetics of LAB were tested in MRS broth at 30 °C. Frozen stock strains were streaked on MRS agar plates, incubated for 48 h and colonies propagated overnight in MRS broth at 30 °C. Then, cells were inoculated at a concentration of about 10^5^ CFU/mL in MRS containing 0%, 2%, 4%, 6% and 8% (*w*/*v*) of NaCl and incubated at 30 °C. Cell turbidity was recorded for 72 h with DEN-1B McFarland densitometer (Biosan, Riga, Latvia) and pH was monitored for the same time with pH meter Basic20 (Crison Instruments, Barcelona, Spain).

Data were modelled using the Gompertz equation as modified by Zwietering et al. [21].

(1) $$y=A\cdot e^{-e^{\left[\left(\frac{e\cdot \mu_{max}}{A}\right)\cdot \left(\lambda -t\right)+1\right]}}$$

When the growth was measured in McFarland units (MF), *y* is the MF value at time t; *A* represents maximum growth expressed as MF value, *µ_max_* is the maximum increase in the growth rate (MF values/h) and *λ* is the time (h) after which MF significantly changes from its baseline. In the case of the pH measurement, the meaning of the parameters was as follows: *y* is the pH decrease at time t; *A* represents the maximum pH decrease; *µ_max_* is the maximum pH decrease rate (ΔpH/h) and *λ* is the lag (h) to detect a measurable pH drop.

### 2.8. Statistical Analysis

The analyses regarding spontaneous fermented sausages characterization were performed in triplicate and the data were statistically analysed using the two-way ANOVA. The Tukey critical difference test was performed to determine differences between samples (*p* < 0.05).

## 3. Results

### 3.1. Spontaneous Fermented Sausages Characterization

The three spontaneous fermented sausages, produced without the use of starter cultures or the addition of sugars to meat batter, were characterized and used as a source of isolation of LAB. Table 3 shows the microbiological parameters, pH and a_w_ of the sausages at the end of ripening.

The products showed high pH values, because of low initial pH decrease due to slow and weak fermentation for the absence of added sugars and starter cultures. Differences among samples can be attributed to the absence, in BAS, of mould growth on the casing, and therefore not contributing to a rise in pH during ripening. The a_w_ was particularly low in the BAS sample (0.831), while it was higher (0.908) in BRE, characterized by a larger diameter.

The microbiological analyses showed a high concentration of LAB in BRE and ROM, with a concentration of about 9 and 8 log CFU/g, respectively. On the contrary, this microbial population was present at a very low concentration (2.54 log CFU/g) in BAS, probably due to the low a_w_ and storage conditions. In BAS, staphylococci and enterococci also showed low concentrations, being enterococci under the detection limit. In BRE and ROM, staphylococci were present at similar levels (about 7 log CFU/g) while enterococci were found in high concentration exclusively in the BRE sample (4.23 log CFU/g). This high number of enterococci could depend on several factors, such as the microbial quality of raw materials, the environment of production or the ripening conditions. The differences in the viable population in BAS can have many reasons. The BAS sample remained one month after ripening under fat, undergoing much longer osmotic stress than other samples that reached final a_w_ values progressively over time during ripening. These stresses can have great influences on microbial populations and their cultivability. In addition, the absence of oxygen in BAS can affect the stress response mechanisms of several bacteria, in particular staphylococci.

### 3.2. Clustering of LAB Isolates and 16S rRNA Sequencing

Representative colonies were picked up from MRS plates deriving from ROM (97 colonies), BRE (50) and BAS (62). After purification, DNA was extracted from each isolate. RAPD fingerprinting and clustering analysis allowed the obtainment of a total of 41 biotypes, as 12 clusters and 10 single strains from ROM, 6 clusters and 6 single strains from BRE and 5 clusters and 5 single strains from BAS (results are shown in the Supplementary Material Figure S1). From this clustering, different scenarios in the spontaneously fermented salami could be observed: (i) dominance of a specific strain in BAS, represented by the cluster containing more than 80% of all isolates; (ii) about 50% single isolate in BRE, with no clear dominance of a particular isolate in the remaining 50%; and (iii) co-dominance of two clusters that comprise more than 50% of the isolates in ROM.

**Figure 1:**
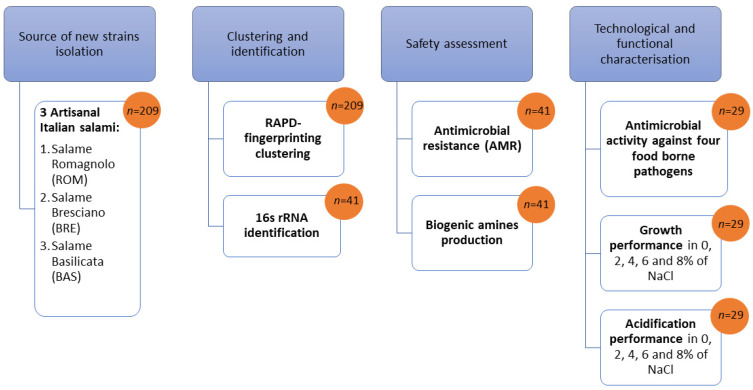
Summary of the screening procedure for isolation and characterization of new potential bioprotective starter lactic acid bacteria (*n* = number of isolates).

A total of 41 biotypes, comprising one representative strain for each cluster and the single strains, showing good growth after sub-culturing, were planned for 16S rRNA sequencing and subsequent analyses (as schematized in Figure 1). Three single isolates present in the clustering profiles (one from BRE and two form ROM) did not show growth after sub-culturing and were discarded. Therefore, the molecular identification was performed on 11 strains from BRE, 20 strains from ROM and 10 strains from BAS (Table 4). Results showed that *Latilactobacillus sakei* was the dominant species (Table 4) in 80.5% of all strains. This confirmed its strong adaptation to the meat matrix and, in particular, to fermented sausages [22,23]. However, within *Lat. sakei*, a great strain biodiversity could be appreciated in all sausages. *Latilactobacillus curvatus* was the second most abundant species among the biotypes, representing 9.1% in BRE, 15.0% in ROM and 20.0% in BAS, whereas *Leuconostoc mesenteroides* was detected only in BAS, representing 20.0% of all isolated strains.

Several authors reported that LAB species diversity in fermented sausages is limited. In fact, *Lat. sakei* predominates during the ripening process, given its competitiveness and assertiveness in the meat matrix, and it was evidenced as the dominant species in some European spontaneously fermented sausages, together with *Lat. curvatus* [24,25]. This predominance is mainly due to its physiological and technological characteristics and to its peculiar metabolic pathways for the meat ecological niche, including the arginine deiminase pathway and the utilization of nucleosides [26,27,28]. *Lat. sakei* can show a high biodiversity degree and, interestingly, strain-specific differences in species performance in meat environments were demonstrated [25].

The present work highlighted the presence of different biotypes in the same sample, but it is known that the co-dominance of these strains can confer competitive exclusion of other strains in the sausage environment and can lead to the peculiar characteristic of the products [29].

The scheme in Figure 1 was used both for the screening procedure and for safety and technological characterization of the studied biotypes.

### 3.3. Determination of Antibiotic Susceptibility (MIC)

The minimal inhibition concentration of eight antibiotics was examined in the 41 LAB biotypes previously identified, following the indications given by EFSA [18]. Table 5 shows the results obtained.

No strains were resistant to gentamycin, streptomycin, erythromycin and clindamycin, while the resistance against the other antibiotics was 14.6% for tetracycline and chloramphenicol, 7.3% for ampicillin and 2.4% for kanamycin.

Resistances to tetracycline and erythromycin are the most widely observed and studied among lactobacilli from fermented meats. Tetracycline is a commonly used antibiotic in pig farming, explaining the abundance of *tet* resistance genes in the pig microbiome and, consequently, in the raw meat used for fermented sausages [30]. In many cases, this specific resistance involved the lactic acid bacteria responsible for meat fermentation; however, the prevalence of resistant strains varied highly in products from different geographical areas [31,32]. In this study, all strains were susceptible to erythromycin and 6 out of 41 (14.6%) were tetracycline resistant. Three of these latter strains were isolated from ROM while the other three were from BAS. According to Fontana et al. [33], 26.4% of *Lat. sakei* and *Lactiplantibacillus plantarum* strains from fermented sausages differing in meat and geographical origin were resistant to tetracycline while the percentage was reduced at 10.7% for erythromycin. Although, according to Abriouel et al. [34], many lactobacilli are intrinsically resistant to kanamycin, streptomycin and gentamicin, in this study, no strains were resistant to streptomycin and gentamicin, and only one strain, isolated from BAS and belonging to *Leuc. mesenteroides* species, was resistant to kanamycin. Danielsen and Wind [35] found higher resistance for *Lat. sakei/curvatus* to kanamycin (66.7%), gentamicin (50%) and streptomycin (100%). Resistant strains were also not found for clindamycin, confirming the data reported by Danielsen and Wind [35] and Fontana et al. [27]. Three ampicillin-resistant strains were found, two from ROM and one from BAS. A higher percentage of resistant strains to this antibiotic were found by Aymerich et al. [36] and by Federici et al. [37].

A higher resistance frequency was observed for chloramphenicol. Five strains out of twenty isolated from ROM were resistant, while only one strain from BRE and none from BAS showed a MIC higher than the cut-off value. Aymerich et al. [36] described a low percentage (1.2%) of *Lat. sakei* resistant to this antibiotic, while Danielsen and Wind [35] found all the strains of *Lat. sakei*/*curvatus* susceptible.

Interestingly, three strains from ROM showed multiple resistances: *Lat. sakei* C12G (tetracycline and chloramphenicol), C22G (tetracycline and ampicillin) and E3G (tetracycline, chloramphenicol and ampicillin). It was demonstrated that genes responsible for tetracycline resistance (*tetM* and *tetS*, which encode ribosomal protection proteins) can mediate the resistance to other antimicrobials such as erythromycin, clindamycin and chloramphenicol [34].

### 3.4. Determination of Biogenic Amines

The 41 identified strains were tested for their ability to decarboxylate tyrosine, histidine, ornithine and lysine. *Lat. curvatus* C26G was the only biogenic amine producer strain. The HPLC analysis of C26G supernatant confirmed the production of putrescine (600 mg/L in Bover–Cid and Holzapfel medium). The production of putrescine by *Lat. curvatus* isolated from dairy products, meat and sausages, ranged from 10 to 10,000 mg/kg as reviewed in the work of Wunderlichova et al. [38]. Putrescine production derives from the decarboxylation of ornithine, an amino acid that can be produced through the arginine deiminase pathway [39]. No strains produced tyramine or histamine, the most dangerous biogenic amine [40,41]. This was expected for *Lat. sakei* strains, while those belonging to *Lat. curvatus* are often involved in tyramine production [42].

### 3.5. Antimicrobial Activity Assay

Twenty-nine strains lacking antimicrobial resistance traits and amino-biogenic potential were included in further screening steps (Figure 1). The LAB culture antagonistic activity against target foodborne pathogens showed that all strains were able to inhibit at least one pathogen to a different extent. Some strains, namely *Lat. sakei* E13G, E15G and C10B, were the most performant antagonistic strains against all four screened pathogens (Table 6). Conversely, non-neutralized and neutralized LAB supernatants did not exert any antimicrobial activity against the same pathogens.

However, two *Lat. sakei* strains, namely C21B and E23B, showed an anti-LAB activity against *Lat. curvatus* DSM 20019^T^ and *Lat. sakei* subsp. *sakei* LMG 13558^T^ in the well diffusion assay (data not shown). Although strain-specific monitoring systems are rarely investigated, a recent study demonstrated how the bacteriogenic strain *Lat. curvatus* TMW 1.624 could exclude some of the *Lat. sakei* strains during sausage fermentation competition, while coexisting in the same environment with bacteriocin resistant *Lat. sakei* 1.417 [43]. The innate resistance against bacteriocin produced by closely related competitors was hypothesised as useful competitive fitness in *Lat. sakei* 23K [26]. Further investigation of bacteriocins in the *Lat. sakei* C21B and *Lat. sakei* E23B genome would elucidate the nature of this anti-LAB activity.

### 3.6. Growth and Acidification Kinetics

**Figure 2:**
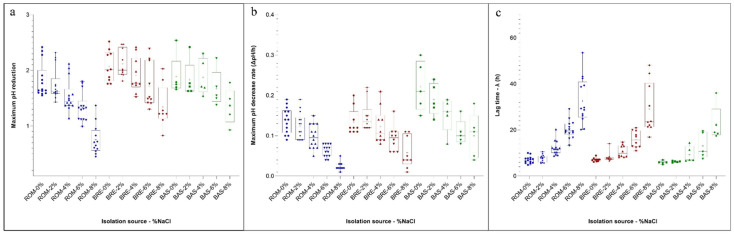
Box and whiskers plots of the parameter estimated for the Gompertz equation of the pH drop curves of strains in relation to the isolation source and NaCl concentration. Boxes represent the limit of the 25th and 75th percentile of the distribution, the line inside the boxes represents the median and the whiskers represent the minimum and maximum values. (**a**) Maximum pH reduction estimates; (**b**) Maximum pH decrease rate estimates (ΔpH/h); (**c**) lag (h) to detect a measurable pH drop. Blue = Salame Romagnolo, Red = Salame Bresciano, Green = Salame Basilicata.

The growth and acidification behaviour of 29 safe LAB strains not presenting antibiotic resistance or amino biogenic potential and studied for their antimicrobial activity, were monitored through the turbidity increase (measured on the McFarland scale) and pH variation under different salt concentrations. The growth data for each strain were modelled with the Gompertz equation [21]. Table S1 and Figure 2 show the biotype growth performances at different NaCl concentrations (0, 2, 4, 6 and 8%). The strains were grouped according to their origin and, for each condition, the estimated parameters of the Gompertz equation (*A*, *λ* and *μ_max_*) were reported as mean (and standard deviation).

The maximum cell density of the McFarland scale (*A*) reached after the incubation was lower, as expected, in the presence of higher salt concentrations. No significant differences were observed in relation to the isolation source in cells grown at 0 and 2% of NaCl. In general, the strains isolated from BRE and BAS showed higher *A* estimates if compared with the strains isolated from ROM. It is noteworthy that comparable standard deviations characterize these data. However, this is not true for the means concerning the *λ* values. In fact, higher NaCl concentration not only determined longer *λ* time but also greater standard deviation of the means, indicating a wide variability in the responses of strains subjected to more severe stress conditions. Analogously to what was observed for *A*, the values of λ did not present significant differences in the samples added with 0 and 2% salt. Shorter λ characterized the strains deriving from BRE and BAS at a higher concentration than those isolated from ROM. The estimates of *λ* followed the same behaviour of *A*, showing higher values for BRE and BAS strains, and were characterized by homogeneous standard deviations. In fermented sausage production, the rate at which starter cultures decrease the pH is crucial for safety and technological aspects [44].

In the same samples analysed for the determination of the McFarland values, pH was also periodically monitored. In fact, the LAB metabolism increased the acidity, and the data were expressed as differences of pH with respect to the initial value. The curve obtained showed a behaviour similar to those obtained measuring the change of the turbidity on the McFarland scale. In this case, the results for the three parameters estimated with the Gompertz equation and obtained in relation to the isolation source and NaCl concentration are presented as a box and whiskers plot (Figure 2a–c).

Regarding the maximum pH decrease (parameter *A* of the model), data showed a great variability within the strains in relation to the source of isolation. In particular, biotypes isolated from ROM were generally characterized by lower pH decreases if compared with BRE and BAS, independently of the NaCl concentration. Even if the extent of the acid production was affected by the NaCl concentration, it is interesting to observe that the variability of the results within each group was of the same order.

Regarding the rate of acidification (the parameter *µ_max_* of the Gompertz equation) (Figure 2b), the strains isolated from BAS presented median values generally higher with respect to the other isolation sources, while the ROM isolates showed lower performances at higher salt concentrations.

Another important characteristic of the starter culture is the time needed to modify the initial pH of the medium (the *λ* value). Also, in this case, the strains isolated from BAS were the more competitive, especially at increasing NaCl concentrations. As observed for the *λ* value for the McFarland scale, the variability within each group markedly increased when the higher content of salts was considered. Highly performant strains could be observed at 6% of NaCl with a short lag phase of 7.5 h. A high heterogeneity among strains can be observed when higher osmotic stress was applied, with a lag phase ranging from 16.92 to 53.55 h. This again confirms the high physiological heterogeneity and potential technological traits of the indigenous meat-borne strains.

The growth and acidification performances of starter cultures are important parameters for ensuring the safety and quality of fermented meats. The rapidity with which the cells can multiply in defined conditions, colonizing the environment and producing organic acids, results in a more efficient inhibition of pathogens or undesirable microbial outgrowth [45]. In addition, in the case of fermented meats, a fast acidification performance represents a crucial technological point allowing the correct initial dehydration process of sausages [46].

The different behaviour shown in the metabolic activity in relation to different NaCl concentrations is often linked to the isolation source. It is noteworthy that the strains isolated from BAS, which were the most performant also at high NaCl concentrations, were those isolated from the harsher environment, whose final a_w_ was 0.831. The great diversity within the species *Lat. sakei* is well known and justifies the differences in adaptation and competition in natural systems, such as fermented sausages [47]. In addition, there can be at least two relationships during meat colonization by members of this species, according to Janßen et al. (2018) [25]. In the first case, a strain outcompetes the other members of microbiota (assertiveness), based on peculiar metabolic characteristics (lag phase, growth rate, antimicrobials, stress responses). Alternatively, a co-dominance or a cooperation between different strains is established, with the consequent creation of a colonization resistance. According to the data reported here, the second strategy characterized the ROM sausages, while BAS and BRE presented a selection of a reduced number of strains at the end of production.

## 4. Conclusions

The present study allowed the selection of autochthonous meat-borne lactic acid bacteria that are safe for their utilization in fermented meat production, effective as starter cultures and promising as bioprotective agents. Some strains isolated from BAS samples showed high growth performances under osmotic pressure and strains C21G, E13G, E15G and C10B showed the greatest antimicrobial activity against *C. sporogenes*, *L. monocytogenes*, *Salmonella* spp. and *E. coli*. These natural isolates have the highest potential as starter cultures and as biopreservative agents in fermented sausage production. The real challenge for future application studies is the use of these microbial cultures to improve the safety of fermented meats, even in situations in which antimicrobial factors, such as the addition of nitrates and nitrites, are reduced or eliminated, as requested by consumers for their potential carcinogenic activity. At the same time, studies concerning the use of autochthonous cultures are pivotal for guaranteeing the specific characteristics and the recognizability of traditional and typical products representing an important cultural heritage.

## Figures and Tables

**Table 1:** Main characteristics of the fermented sausages used for the isolation of LAB strains.

	*Salame Romagnolo* (ROM)	*Salame Bresciano* (BRE)	*Salame Basilicata* (BAS)
Diameter (mm)	55	80	35
Final weight (g)	800	500	250
% NaCl (*w*/*w* in the batter)	2.8	2.4	2.5
Nitrate/nitrite addiction	Not added	KNO_3_ (0.015%)	Not added
Fat in the meat batter (%)	20 (with lard cube)	25 (minced with lean meat)	25 (minced with lean meat)
Ripening time (days)	60	75	30
Weight loss (%)	35	30	35
Casing	Natural	Natural	Natural
Presence of moulds on casing	Yes	Yes	No

**Table 2:** Indicator strains used for the in vitro antimicrobial activity.

Indicator Strains Used in the Assays	Cultivation Conditions	Strains Used in the Well-Diffusion Assay (WDA)	Strains Used in the Spot Test
*Clostridium sporogenes*	RCM, 37 °C, 24–48 h, anaerobic	+	+
*Listeria innocua* DPC 3572	BHI, 37 °C, 24 h	+	+
*L. monocytogenes* DPC 1768	BHI, 37 °C, 24 h	+	+
*Escherichia coli* DPC 6054	BHI, 37 °C, 24 h	+	+
*Salmonella enterica* ssp. Typhimurium DPC 6046	BHI, 37 °C, 24 h	+	+
*Latilactobacillus curvatus* DSM 20019^T^	MRS, 30 °C, 24–48 h	+	−
*Latilactobacillus sakei* ssp. *sakei* LMG 13558 ^T^	MRS, 30 °C, 24–48 h	+	−
*Lactobacillus delbrueckii* ssp. *bulgaricus* LMG 6901^T^	MRS, 37 °C, 24–48 h, anaerobic	+	−
*Lactiplantibacillus plantarum* DPC6124	MRS, 37 °C, 24–48 h,	+	−
*Lacticaseibacillus paracasei* ssp. *paracasei* DPC6130	MRS, 30 °C, 24–48 h	+	−
*Levilactobacillus brevis* LMG 6906^T^	MRS, 37 °C, 24–48 h, anaerobic	+	−
*Limosilactobacillus fermentum* DPC 6193	MRS, 37 °C, 24–48 h, anaerobic	+	−

Strain was used (+) or not used (−) in the assay.

**Table 3:** Microbiological counts and chemico-physical features of the spontaneously fermented sausages at the end of ripening.

	Sample ROM	Sample BRE	Sample BAS
Lactic acid bacteria (log CFU/g)	7.79 ^b^	9.09 ^a^	2.54 ^c^
Staphylococci (log CFU/g)	7.09 ^b^	6.75 ^a^	2.52 ^c^
Enterococci (log CFU/g)	<1 ^b^	4.23 ^a^	<1 ^b^
pH	6.01 ^b^	6.19 ^a^	5.86 ^b^
a_w_	0.853 ^b^	0.908 ^a^	0.831 ^c^

Different lower-case letters represent statistically significant differences (*p* < 0.05) between samples according to Tukey test of the two-way ANOVA.

**Table 4:** Identification of lactobacilli isolated from three spontaneously fermented sausages.

Isolation Source	Strain	Closest Match	% Identification NCBI	Accession Number
Salame Romagnolo (ROM)	C10G	*Lat. sakei*	99.858	MN173314
	C12G	*Lat. sakei*	99.929	MN173315
	C16G	*Lat. sakei*	99.721	MN173316
	C21G	*Lat. sakei*	100.000	MW548285
	C22G	*Lat. sakei*	99.857	MN173317
	C26G	*Lat. curvatus*	99.785	MN173318
	C27G	*Lat. sakei*	99.929	MN173319
	C45G	*Lat. sakei*	99.786	MN173320
	C48G	*Lat. sakei*	100.000	MN173321
	E1G	*Lat. sakei*	99.786	MN173322
	E3G	*Lat. sakei*	99.929	MN173323
	E8G	*Lat. curvatus*	99.645	MN173324
	E13G	*Lat. sakei*	99.857	MN173325
	E15G	*Lat. sakei*	99.929	MN173326
	E17G	*Lat. sakei*	99.786	MN173327
	E18G	*Lat. curvatus*	99.856	MN173328
	E19G	*Lat. sakei*	100.000	MN173329
	E22G	*Lat. sakei*	99.588	MW548286
	E26G	*Lat. sakei*	99.929	MN173330
	E28G	*Lat. sakei*	99.929	MN173331
Salame Bresciano (BRE)	C3B	*Lat. sakei*	99.857	MN120894
	C10B	*Lat. sakei*	99.786	MN173305
	C14B	*Lat. sakei*	99.929	MN173306
	C16B	*Lat. sakei*	99.929	MN173307
	C17B	*Lat. sakei*	99.857	MN173308
	C21B	*Lat. sakei*	99.930	MN173309
	C22B	*Lat. sakei*	99.859	MN173310
	E3B	*Lat. sakei*	99.860	MN173311
	E7B	*Lat. curvatus*	99.786	MN173312
	E15B	*Lat. sakei*	99.930	MN173313
	E23B	*Lat. sakei*	99.616	MN215967
Salame Basilicata (BAS)	BC1	*Leuc. mesenteroides*	99.930	MN173332
	BC6	*Lat. sakei*	99.930	MN173333
	BC20	*Lat. sakei*	99.930	MN173334
	BC33	*Lat. sakei*	99.930	MN173335
	BC35	*Lat. sakei*	100.000	MN173336
	BC50	*Lat. sakei*	99.929	MN173337
	BE2	*Lat. curvatus*	99.788	MN173338
	BE16	*Lat. sakei*	100.000	MN173339
	BE23	*Leuc. mesenteroides*	100.000	MN173340
	BE28	*Lat. curvatus*	99.861	MN173341

**Table 5:** Phenotypic characterization of the antibiotic resistance in LAB meat-borne strains previously identified.

			Antibiotic Minimal Inhibitory Concentration (µg/mL)							
Isolation Source	Species	Strains	Gen	Kan	Str	Tet	Ery	Cli	Chl	Amp
Salame Romagnolo (ROM)	*Lat. sakei*	C10G	1 (S) ^a^	8 (S)	16 (S)	4 (S)	0.12 (S)	0.06 (S)	4 (S)	2 (S)
	*Lat. sakei*	C12G	1 (S)	8 (S)	16 (S)	16 (R) ^b^	0.12 (S)	0.25 (S)	8 (R)	2 (S)
	*Lat. sakei*	C16G	<0.5 (S)	<2 (S)	8 (S)	4 (S)	0.06 (S)	0.03 (S)	4 (S)	1 (S)
	*Lat. sakei*	C21G	<0.5 (S)	<2 (S)	8 (S)	4 (S)	0.06 (S)	0.03 (S)	4 (S)	1 (S)
	*Lat. sakei*	C22G	1 (S)	16 (S)	32 (S)	>64 (R)	0.12 (S)	0.25 (S)	4 (S)	>16 (R)
	*Lat. curvatus*	C26G	2 (S)	32 (S)	16 (S)	1 (S)	0.06 (S)	0.03 (S)	8 (R)	0.5 (S)
	*Lat. sakei*	C27G	<0.5 (S)	4 (S)	8 (S)	4 (S)	0.12 (S)	0.03 (S)	4 (S)	2 (S)
	*Lat. sakei*	C45G	2 (S)	16 (S)	32 (S)	8 (S)	0.12 (S)	0.03 (S)	4 (S)	2 (S)
	*Lat. sakei*	C48G	1 (S)	8 (S)	16 (S)	8 (S)	0.12 (S)	0.06 (S)	8 (R)	4 (S)
	*Lat. sakei*	E1G	<0.5 (S)	4 (S)	16 (S)	8 (S)	0.12 (S)	0.03 (S)	4 (S)	1 (S)
	*Lat. sakei*	E3G	2 (S)	16 (S)	32 (S)	16 (R)	0.12 (S)	0.5 (S)	8 (R)	>16 (R)
	*Lat. curvatus*	E8G	1 (S)	8 (S)	8 (S)	2 (S)	0.12 (S)	0.12 (S)	8 (R)	1 (S)
	*Lat. sakei*	E13G	1 (S)	8 (S)	64 (S)	8 (S)	0.12 (S)	0.06 (S)	4 (S)	2 (S)
	*Lat. sakei*	E15G	<0.5 (S)	<2 (S)	4 (S)	2 (S)	0.12 (S)	0.03 (S)	2 (S)	2 (S)
	*Lat. sakei*	E17G	<0.5 (S)	4 (S)	8 (S)	8 (S)	0.12 (S)	0.06 (S)	4 (S)	2 (S)
	*Lat. curvatus*	E18G	<0.5 (S)	<2 (S)	4 (S)	4 (S)	0.06 (S)	0.06 (S)	2 (S)	0.5 (S)
	*Lat. sakei*	E19G	1 (S)	8 (S)	16 (S)	4 (S)	0.12 (S)	0.06 (S)	4 (S)	1 (S)
	*Lat. sakei*	E22G	1 (S)	4 (S)	16 (S)	4 (S)	0.25 (S)	0.06 (S)	4 (S)	1 (S)
	*Lat. sakei*	E26G	<0.5 (S)	<2 (S)	8 (S)	4 (S)	0.06 (S)	0.03 (S)	4 (S)	1 (S)
	*Lat. sakei*	E28G	0.5 (S)	<2 (S)	4 (S)	8 (S)	0.12 (S)	0.03 (S)	4 (S)	1 (S)
Salame Bresciano (BRE)	*Lat. sakei*	C3B	1 (S)	4 (S)	16 (S)	4 (S)	0.12 (S)	0.12 (S)	8 (R)	2 (S)
	*Lat. sakei*	C10B	1 (S)	8 (S)	16 (S)	2 (S)	0.25 (S)	0.03 (S)	2 (S)	2 (S)
	*Lat. sakei*	C14B	1 (S)	8 (S)	16 (S)	4 (S)	0.12 (S)	0.06 (S)	1 (S)	2 (S)
	*Lat. sakei*	C16B	<0.5 (S)	<2 (S)	8 (S)	4 (S)	0.12 (S)	0.06 (S)	2 (S)	0.5 (S)
	*Lat. sakei*	C17B	1 (S)	4 (S)	16 (S)	4 (S)	0.5 (S)	0.25 (S)	4 (S)	2 (S)
	*Lat. sakei*	C21B	<0.5 (S)	4 (S)	16 (S)	4 (S)	0.12 (S)	0.12 (S)	2 (S)	4 (S)
	*Lat. sakei*	C22B	2 (S)	4 (S)	1 (S)	4 (S)	0.25 (S)	0.06 (S)	4 (S)	4 (S)
	*Lat. sakei*	E3B	<0.5 (S)	<2 (S)	8 (S)	4 (S)	0.12 (S)	0.06 (S)	2 (S)	0.5 (S)
	*Lat. curvatus*	E7B	<0.5 (S)	<2 (S)	8 (S)	2 (S)	0.25 (S)	0.06 (S)	1 (S)	2 (S)
	*Lat. sakei*	E15B	1 (S)	4 (S)	16 (S)	4 (S)	0.12 (S)	0.06 (S)	2 (S)	2 (S)
	*Lat. sakei*	E23B	0.5 (S)	2 (S)	8 (S)	8 (S)	0.12 (S)	0.06 (S)	4 (S)	2 (S)
Salame Basilicata (BAS)	*Leuc. mesenteroides*	BC1	4 (S)	64 (R)	1 (S)	2 (S)	0.12 (S)	0.12 (S)	4 (S)	2 (S)
	*Lat. sakei*	BC6	8 (S)	1 (S)	32 (S)	>64 (R)	0.12 (S)	0.5 (S)	2 (S)	2 (S)
	*Lat. sakei*	BC20	4 (S)	1 (S)	32 (S)	2 (S)	0.12 (S)	0.25 (S)	2 (S)	2 (S)
	*Lat. sakei*	BC33	2 (S)	8 (S)	1 (S)	2 (S)	0.12 (S)	0.03 (S)	4 (S)	4 (S)
	*Lat. sakei*	BC35	4 (S)	1 (S)	32 (S)	64 (R)	0.12 (S)	0.5 (S)	2 (S)	2 (S)
	*Lat. sakei*	BC50	2 (S)	8 (S)	32 (S)	2 (S)	0.12 (S)	0.03 (S)	4 (S)	2 (S)
	*Lat. curvatus*	BE2	2 (S)	1 (S)	1 (S)	4 (S)	0.12 (S)	0.03 (S)	4 (S)	4 (S)
	*Lat. sakei*	BE16	4 (S)	32 (S)	64 (S)	4 (S)	0.12 (S)	0.03 (S)	4 (S)	8 (R)
	*Leuc. mesenteroides*	BE23	2 (S)	8 (S)	1 (S)	32 (R)	0.12 (S)	0.06 (S)	2 (S)	2 (S)
	*Lat. curvatus*	BE28	1 (S)	8 (S)	1 (S)	1 (S)	0.12 (S)	0.03 (S)	2 (S)	1 (S)

Abbreviation of antibiotics: Gen = Gentamicin, Kan = Kanamycin, Str = Streptomycin, Tet = Tetracyclin, Ery = Erythromycin, Cli = Clindamycin, Chl = Chloramphenicol, Amp = Ampicillin, ^a^: S = sensitive (≤cut off); ^b^: R (Marked in red) = resistant (>cut off).

**Table 6:** Spot-test assay: direct antagonistic activity of meat-borne LAB against foodborne pathogens.

Isolation Source	Species	Strains	*C. sporogenes*	*L. monocytogenes*	*Salmonella* spp.	*E. coli*
Salame Romagnolo (ROM)	*Lat. sakei*	C10G				
	*Lat. sakei*	C16G				
	*Lat. sakei*	C21G				
	*Lat. sakei*	C27G				
	*Lat. sakei*	C45G				
	*Lat. sakei*	E1G				
	*Lat. sakei*	E13G				
	*Lat. sakei*	E15G				
	*Lat. sakei*	E17G				
	*Lat. sakei*	E18G				
	*Lat. sakei*	E19G				
	*Lat. sakei*	E22G				
	*Lat. sakei*	E26G				
	*Lat. sakei*	E28G				
Salame Bresciano (BRE)	*Lat. sakei*	C10B				
	*Lat. sakei*	C14B				
	*Lat. sakei*	C16B				
	*Lat. sakei*	C17B				
	*Lat. sakei*	C21B				
	*Lat. sakei*	C22B				
	*Lat. sakei*	E3B				
	*Lat. curvatus*	E7B				
	*Lat. sakei*	E15B				
	*Lat. sakei*	E23B				
Salame Basilicata (BAS)	*Lat. sakei*	BC20				
	*Lat. sakei*	BC33				
	*Lat. sakei*	BC50				
	*Lat. curvatus*	BE2				
	*Lat. curvatus*	BE28				

Different colours indicate different inhibition level: white = no inhibition, yellow = weak inhibition, orange = medium inhibition, red = strong inhibition.

## Data Availability

The datasets generated during and/or analysed during the current study are available from the corresponding author upon reasonable request.

## Supplementary Materials

The following supporting information can be downloaded at: https://www.mdpi.com/article/10.3390/foods12040727/s1, Figure S1: RAPD-profiles of 209 autochthonous lactic acid bacteria, isolated from three artisanal Italian salami; Table S1: Mean values (±standard deviation) of the parameters *A* (Mc Farland value), *λ* (h) and *μ_max_* (h^−1^) of the growth curves obtained by the modelization with the Gompertz equation, for strains isolated from the different salami at different NaCl concentrations.
