# Supplementary material for: Characterization of Lactic Acid Bacteria Isolated from Spontaneously Fermented Sausages: Bioprotective, Technological and Functional Properties
Source: Foods. 2023 Feb 7;12(4):727. doi: 10.3390/foods12040727 (PMC9955731; doi:10.3390/foods12040727)
Supplement: Supplementary file 1 [file foods-12-00727-s001.zip › foods-2184212-supplementary.pdf]

Supplementary material

Figure S1. RAPD-profiles of 209 autochthonous lactic acid bacteria, isolated from three artisanal Italian salami.

(A). RAPD-PCR fingerprinting: Salame Romagnolo (ROM)

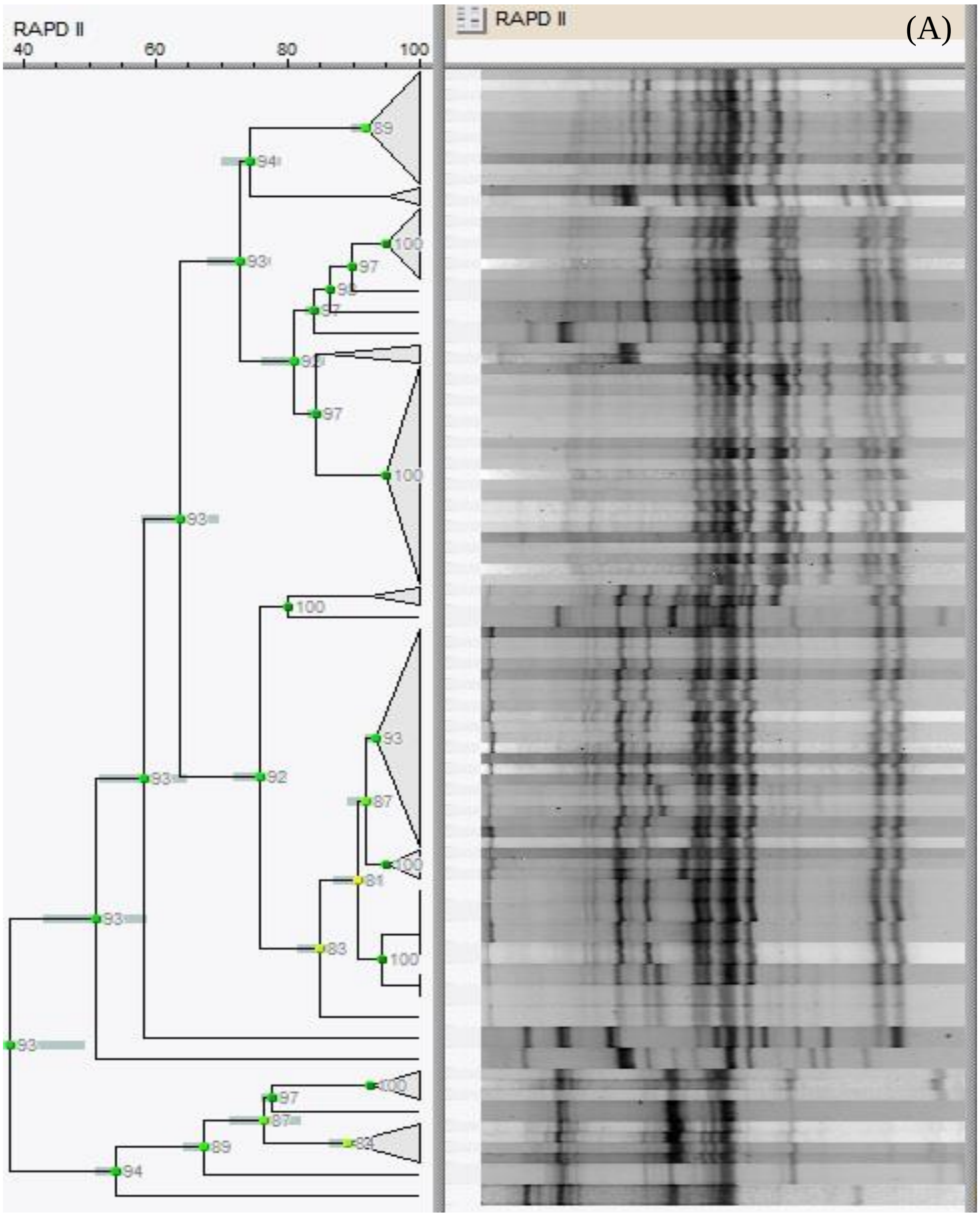

(B). RAPD-PCR fingerprinting: Salame Bresciano (BRE)

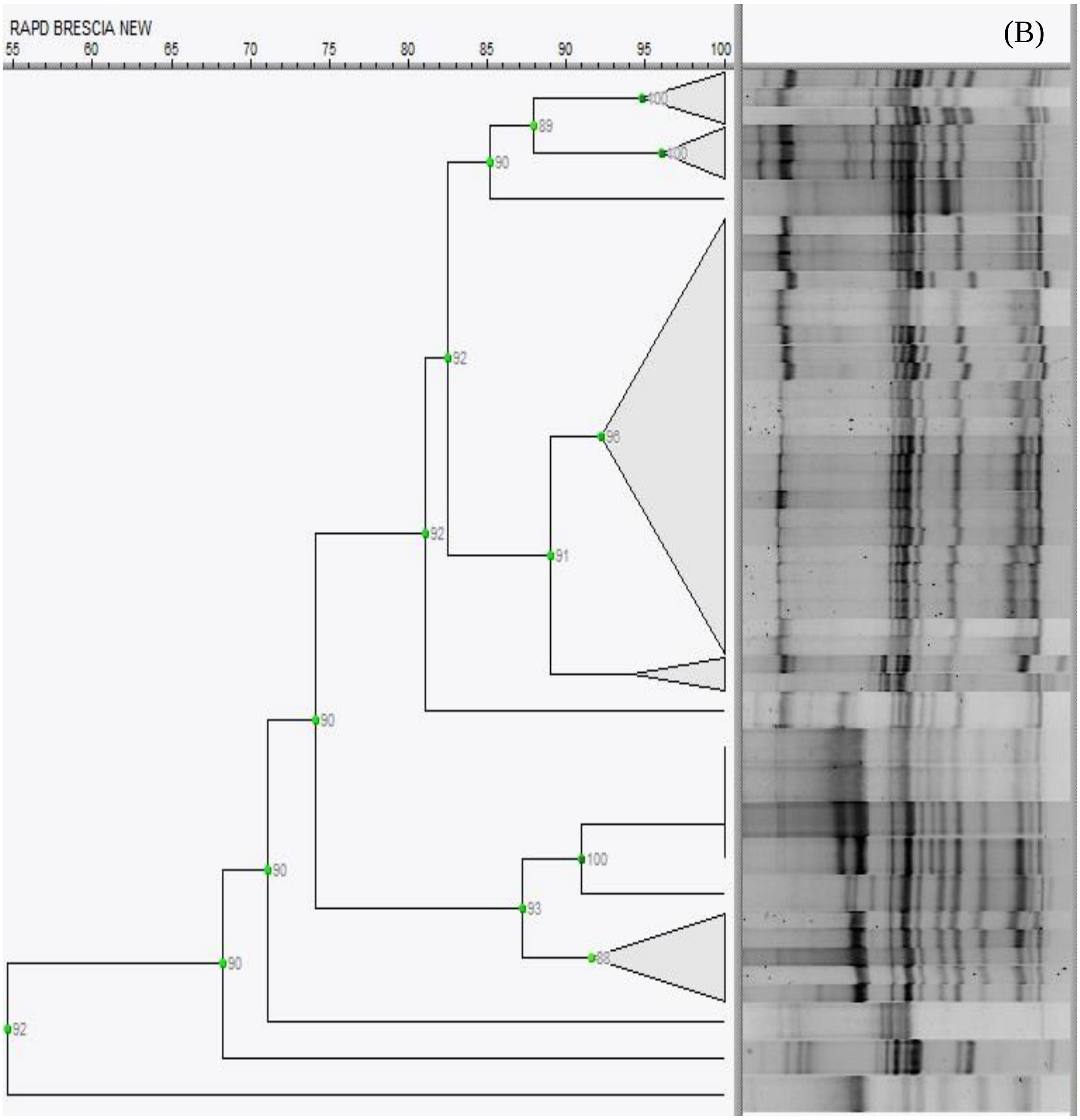

(C). RAPD-PCR fingerprinting: Salame Basilicata (BAS)

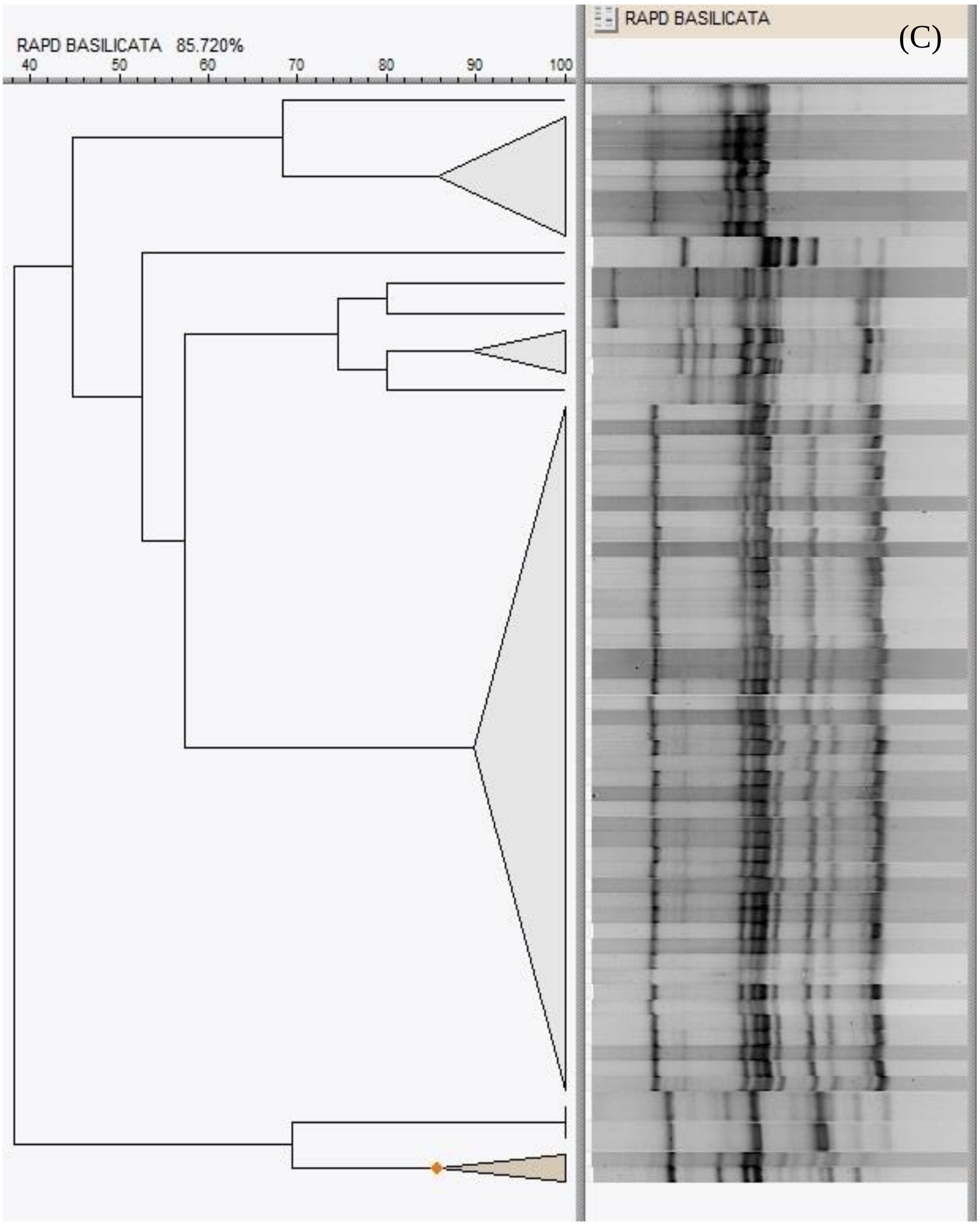

Table S1. Mean values (± standard deviation) of the parameters *A* (Mc Farland value), *λ* (h) and μ_{max} (h^{-1}) of the growth curves obtained by the modelling with the Gompertz equation, for strains isolated from the different salami at different NaCl concentrations. Different lower-case letters represent statistically significant differences (p<0.05) between values in the same column.

Isolation source	%NaCl	*A* (Mc Farland)	*λ* (h)	μ_{max} (h^{-1})
Salame Romagnolo (ROM) *n*=14 strains	0	8.89 (±1.18)[a]	7.08 (±1.51)[ab]	0.72 (±0.12)[bc]
	2	8.13 (±1.27)[abc]	7.67 (±1.55)[ab]	0.6 (±0.07)[cd]
	4	6.79 (±1.38)[de]	12.18 (±3.06)[cd]	0.53 (±0.01)[d]
	6	5.57 (±0.68)[f]	20.23 (±3.9)[e]	0.38 (±0.12)[e]
	8	3.43 (±1.08)[h]	31.72 (±9.9)[g]	0.14 (±0.06)[f]
Salame Bresciano (BRE) *n*=10 strains	0	8.76 (±1.24)[ab]	7.12 (±0.79)[ab]	0.94 (±0.24)[a]
	2	8.23 (±0.99)[abc]	8.18 (±2.13)[ab]	0.95 (±0.19)[a]
	4	7.47 (±0.86)[cd]	10.77 (±2.58)[bc]	0.6 (±0.03)[cd]
	6	6.13 (±0.84)[ef]	15.88 (±3.62)[d]	0.4 (±0.17)[e]
	8	4.50 (±1.11)[g]	25.92 (±9.72)[f]	0.3 (±0.37)[e]
Salame Basilicata (BAS) *n*=5 strains	0	8.76 (±0.72)[ab]	5.96 (±0.65)[a]	0.98 (±0.12)[a]
	2	8.44 (±0.50)[abc]	6.31 (±0.30)[ab]	0.85 (±0.24)[ab]
	4	7.61 (±0.68)[bcd]	9.74 (±3.31)[abc]	0.66 (±0.05)[bcd]
	6	6.68 (±0.70)[de]	14.18 (±5.65)[cd]	0.59 (±0.14)[cd]
	8	5.32 (±1.42)[fg]	22.85 (±6.93)[ef]	0.35 (±0.24)[e]
